# Accelerated Shelf-Life and Stability Testing of Hydrolyzed Corn Starch Films

**DOI:** 10.3390/polym15040889

**Published:** 2023-02-10

**Authors:** Andra-Ionela Ghizdareanu, Diana Pasarin, Alexandra Banu, Andreea Ionita (Afilipoaei), Cristina Emanuela Enascuta, Alexandru Vlaicu

**Affiliations:** 1 Facultatea de Stiinta si Ingineria Materialelor, Universitatea Politehnica din Bucuresti, 313 Splaiul Independentei, 060042 Bucharest, Romania; 2 Institutul National de Cercetare-Dezvoltare pentru Chimie si Petrochimie, ICECHIM, 202 Splaiul Independentei, 060021 Bucharest, Romania

**Keywords:** enzymatic hydrolysis, corn starch hydrolysate, food packaging films, shelf life, stability, biodegradability

## Abstract

Nonbiodegradable food packaging films are made from plastics such as polyethylene and polypropylene, which can take hundreds of years to decompose and create environmental hazards. On the other hand, biodegradable food packaging films are made from renewable materials such as corn starch or cellulose, that degrade within a few weeks or months and prove to be more sustainable and environmentally friendly. In this work, we used corn starch hydrolyzed (CSH) with α-amylase to prepare a film with biodegradable properties. The film was tested for 60 days at different accelerated temperatures and relative humidity (RH), 13 ± 2 °C and 65 ± 5% RH, 23 ± 2 °C and 45 ± 5% RH, and 33 ± 2 °C and 30 ± 5% RH, to test its durability and stability. Soil biodegradation of the CSH film was evaluated by visual appearance, microscopic observation, weight loss, scanning electron microscopy (SEM), and Fourier-transformed infrared spectroscopy (FTIR) every 6 days. The film was found to have strong hygroscopic properties and was able to last up to 10 months if it is maintained at 20 ± 5 °C and 45 ± 5% RH. After the biodegradability test for at least 30 days, the film showed a significantly higher weight loss rate and microbial activity on the surface of the film, which indicates that the film is biodegradable. The present work recommends biodegradable CSH films as an excellent environmentally friendly choice for dried foods packaging, due to their good shelf life at room temperature, which is beneficial when shipping and storing products, but these films are not suitable for foods with high moisture content.

## 1. Introduction

The overuse of plastic packaging films and the accumulation of plastic waste due to the long degradation period have increased the interest in environmentally friendly packaging films from natural and sustainable sources [1]. Polymeric materials are used extensively throughout the world. Due to their properties and capabilities, these materials have a significant advantage over other, more traditional materials such as metals and wood; it is believed that 99% of these polymeric materials are derived from fossil fuels. Since their main raw material poses a threat to the preservation of the environment, these plastics pose a number of problems. The degradability and durability of these materials are incompatible. For most applications, it is beneficial if the material retains certain properties over a long period of time, but it is also desirable that it can be easily disposed of and degraded after use [2]. Currently, films used for food packaging are not easily biodegradable in soil or by microorganisms. To promote plastic decomposition and reduce soil C and N_2_O losses under field conditions, N fertilization may be advisable in conjunction with optimal irrigation (to ensure adequate redox conditions) [3].

Therefore, plant-derived natural polymers are considered a promising alternative to replace synthetic packaging [4]. In recent years, research has been conducted in various fields to develop biodegradable materials for different applications. Recently, a dextran hybrid hydrogel based on peptide nanopods was produced. The combination of all these compounds makes it a promising new biomaterial platform for expanding its applications in tissue engineering and drug delivery [5]. The potential applications of biomaterials inspired by membraneless organelles (MO), with examples from biochemical reactors, synthetic biology, drug discovery, and drug delivery, were also discussed [6]. Due to their excellent biocompatibility and degradability, natural substances such as cationic polysaccharides and (poly)peptides have recently attracted great interest in the construction of novel polymeric vectors [7]. Biodegradable packaging films based on starch from different sources have gained attention in recent years due to their large occurrence in nature, non-toxicity, film-forming ability, and biodegradability [8]. The fact that starch is composed of two different carbohydrate polymers, namely amylose (20–30%) and amylopectin (70–80%), which are extremely high molecular weight polymers, has increased the interest in starch as the most important film-forming product in the packaging industry [9]. It has been demonstrated that cassava bagasse can be used effectively as a non-edible starch-based material for film production. In order to determine if the produced films are suitable for commercial biodegradable food packaging, additional research is needed to look into the mechanical and functional properties, food security, and biodegradability of the films [10].

Since the starch sources have different amylose/amylopectin ratios, the film-forming properties are also different [11]. Higher amylose content is preferable to obtain an opaque and thicker film, whereas thinner and transparent films are obtained with lower amylose content. While the amylopectin content provides the optimum consistency of the starch film solution, amylose determines the optical properties of the film [12]. However, the use of starch to develop packaging films is associated with several drawbacks, such as poor thermal and moisture stability [13]. To improve the mechanical properties of starch-based films, Sommerfield et al. [14] reduced the average molecular weight and amylopectin content of corn and potato starch by partial hydrolysis using acid catalysis and obtained readily biodegradable films. Zhang H et al. [15] increased pea starch films’ tensile strength and relative crystallinity by acid hydrolysis of pea starch with hydrochloric acid. In the study by Martins et al. [16], films were prepared from acid-hydrolyzed (hydrochloric acid) and esterified rice starch (citric acid), which exhibited higher tensile strength and relative crystallinity and better biodegradability, but lower water solubility. Although starch is not thermoplastic, it can be extruded or injected to produce films, similar to other polymers, after the addition or in the presence of plasticizers such as glycerol or water [17].

To improve the film-forming properties, the polymeric starch matrix can be modified or reinforced with various agents or other polymers. The final properties of starch-based films also depend on the processing technique or the type of plasticizer used [18]. The addition of plasticizers improves films’ flexibility and processing properties, but at higher levels reduces moisture inhibition and flexibility [19]. Studies have shown that the incorporation of glycerin at high concentrations in films based on arrowroot starch (Maranta arundinaceous) increases film thickness, moisture content, and water solubility [20].

Moisture transfer in packaged foods can lead to their spoilage and depends mainly on the water activity in the food, the temperature and humidity conditions in the storage environment, but also the moisture of the packaging and its permeability to water vapor; this phenomenon is of great importance to the food packaging industry [21]. The water permeability of starch-based films is a complex phenomenon due to the strong interaction of water molecules with the polymeric matrix of starch.

The water sorption isotherms of starch-based films are highly nonlinear in the range of 5–45 °C due to the varying amylose content of the starches. In general, the main mechanical properties of these water-absorbent polymers are highly dependent on their water content and ambient relative humidity [22]. Therefore, the determination of the shelf life and stability of food packaging films is highly dependent on the permeability and moisture absorption properties. The moisture absorption of the packaging is particularly important when packaging a moisture-sensitive food, and all phenomena affecting this must be taken into account [23]. In the previous study, the data for two different mathematical models (Q10 rule and Arrhenius equation) were relatively similar. The data were extrapolated to real temperature to analyze the influence of accelerated temperature conditions on fruit smoothies [24].

The shelf life of moisture-sensitive products can be predicted using Arrhenius’ mathematical model by considering the moisture content, deterioration over time, and permeability characteristics of the package concerning the relative humidity and temperature of the storage environment.

Due to the limited time, due to the long shelf life of food packaging, predictions of quality deterioration as a function of important parameters along the food chain, such as moisture, temperature, or UV radiation, are often made using mathematical models [25].

The aim of this study was to estimate the shelf life at ambient temperature and under normal RH conditions by accelerated stability tests, and to investigate the biodegradability of food packaging films produced from enzymatically hydrolyzed corn starch.

## 2. Materials and Methods

Corn starch was purchased from SCM Colin Daily Romania, and glacial acetic acid and glycerol were purchased from SC Chimreactiv SRL. α-amylase, from Bacillus amyloliquefaciens (A7595), was purchased from Sigma-Aldrich (St. Louis, MO, USA). All the materials were food-grade ingredients.

### 2.1. Preparation of Corn Starch Hydrolysate (CSH)

Enzymatic hydrolysis of corn starch with α-amylase from Bacillus amyloliquefaciens was used to prepare biofilms for food packaging, based on the method described by Kong et al. [26] with minor modifications.

The polymeric matrix of hydrolyzed corn starch was reinforced by the addition of glycerol as a plasticizer. A material commonly used for food packaging, polyethylene, was selected for biodegradability comparisons.

### 2.2. Preparation of CSH Films

CSH films were prepared using the casting method described by Beer-Lech et al. [27] using CSH, glycerol, and glacial acetic acid (as crosslinking agents). The CSH film-forming solution was prepared by mixing 20 g of CSH with 150 mL of distilled water at 50 °C.

To the film-forming solution, 10 mL of glycerol as plasticizer (1:2 *v*/*w* of CSH) and 10 mL of 0.5 M acetic acid were added. The mixture was kept at 70 °C with a heating magnetic stirrer under constant stirring at 300 rpm until complete gelatinization.

The film-forming solution was then cooled to 50 °C and poured into glass plates (20 × 20 cm) to ensure that the film thickness was uniform, and dried at room temperature of 25 °C for about 72 h. After drying, the film was removed from the glass plates and stored in a desiccator until characterization.

### 2.3. Physical and Hygroscopic Properties: Film Thickness, Moisture Content (Mc), Swelling Degree (Sd), and Total Soluble Matter (Tsm)

The hygroscopic properties of film-forming materials are considered important factors in choosing films for use in specific applications. Water resistance and integrity are required for packaging foods with high moisture content, so water solubility or moisture absorption of the material used may be detrimental. The CSH film samples were compared in terms of their hygroscopic properties to a control sample obtained with non-hydrolyzed corn starch.

#### 2.3.1. Film Thickness

The film thickness was measured in five points (one in the center and the others in different parts of the film) using a digital micrometer. The average thickness value was calculated.

#### 2.3.2. Moisture Content (*Mc*), Swelling Degree (*Sd*), and Total Soluble Matter (*Tsm*)

*Mc*, *Sd*, and *Tsm* were determined according to the three-step method described by Janik W. et al. [28] with minor modifications. For *Mc*, *Sd*, and *Tsm* determination, the weights *M*1, *M*2, *M*3, and *M*4 were determined. Triplicate specimens of CSH film samples and the control sample were cut into pieces 3 × 3 cm and weighed on an analytical balance with an accuracy of 0.001 (*M*1). The samples were dried for 24 h at 105 °C and weighed again (*M*2). Then, the CSH film samples and the control samples were kept in 50 mL of distilled water for approximately 24 h and weighed again (*M*3). For the determination of *Tsm*, the CSH film samples and the control samples were dried again for 24 h at 105 °C and weighed (*M*4). All measurements were performed at least three times, and the mean values were calculated using the following equations (Equations (1)–(3)):

(1) $$Mc=\frac{M1-M2}{M2}\times 100,$$

(2) $$Sd=\frac{M3-M2}{M2}\times 100,$$

(3) $$Tsm=\frac{M2-M4}{M2}\times 100$$

#### 2.3.3. Water Vapor Permeability (*WVP*)

The water barrier efficiency of films is indicated by the *WVP* value and is an important factor to monitor because it can affect the quality of food packaged in films, especially during distribution and storage [29]. The predominance of hydrophilic molecules such as glycerol and the hydrophilic structure of starch, which constitute the majority of compounds in the matrix of CSH film, facilitate the diffusion of water molecules inward; therefore, the *WVP* of CSH films depends on the hydrophobic–hydrophilic balance of the components that make up the films and the degree of bonding between the molecules [30].

The *WVP* value for the CSH film samples was determined according to the method described by Kumar et al. [31,32] and according to ASTM E96 [33] with minor modifications. Briefly, Berzelius beakers containing calcium chloride (pre-dried at 105 ± 1 °C for 24 h) were sealed with CSH film samples placed on the top of the beaker. All Berzelius beakers covered with CSH film samples were weighed and placed in a desiccator where the relative humidity was maintained at 75% RH (using the saturated solution of sodium chloride, NaCl). The temperature and RH inside and outside the desiccator were recorded using a digital instrument for measuring temperature and humidity.

The desiccator was placed in an incubator at 35 ± 3 °C. The weight of all beakers was recorded every 8 h for the first 24 h and every 24 h thereafter for 3 days. All measurements were performed at least three times for each sample. *WVP* was calculated using Equation (4) after plotting the weight gain of the samples as a function of time:

(4) $$WVP=\left(\frac{Slope}{A}\times t\right)/\Delta P,$$

where the slope of the line was calculated by linear regression (R^2^ > 0.9) of the weight change versus time, *t* is the average film thickness (mm), *A* is the exposed film area (mm^2^) and *ΔP* is the partial vapor pressure difference between the outside and inside of the film-covered beaker (RH 75%–RH 0%), *ΔP* = 0.35 kPa.

### 2.4. Accelerated Shelf Life and Stability Testing

The accelerated shelf-life testing (ASLT) method was applied to obtain rapid data, which were modeled and statistically analyzed to provide information on the shelf life of CSH film under normal conditions of use and storage. The ASLT method consists of keeping the samples under extreme storage conditions to accelerate their degradation and constantly monitor the parameters with direct influence on their shelf life. The CSH film samples were tested according to the protocols for the ASLT method described by Mizrahi et al. [34].

The ASLT method applies to any degradation process (mechanical, chemical, physical, biochemical, or microbiological) for which there is a valid kinetic model and experimental data that can be extrapolated [35]. To predict the actual shelf life, it is necessary to evaluate how the deterioration process behaves as a function of time under accelerated conditions [36].

The method can be achieved by two different approaches, either applied separately or simultaneously:

- Time compression (high usage rate, testing is performed more intensively than actual usage);
- Accelerated degradation testing (the same conditions that degrade the product under normal storage conditions).

Variables that can accelerate the degradation of packaging films are usually temperature, humidity, pH, light, or UV rays [37,38,39].

For CSH film samples, the hygroscopic depreciation and folding strength were monitored during the test period to observe how different extreme storage conditions influence the stability and shelf life.

Triplicate specimens of CSH film samples were placed at three different temperatures and RH conditions (13 ± 2 °C and 65 ± 5% RH, 23 ± 2 °C and 45 ± 5% RH, and 33 ± 2 °C and 30 ± 5% RH) in climate chambers. The samples were removed from the chambers at a 15 day interval to determine the moisture absorption and folding strength.

#### 2.4.1. Moisture Absorption (*Ma*)

*Ma* was determined according to the method described in ASTM D 570-98 [40]. Triplicate specimens of CSH film samples were cut into pieces 3 × 3 cm, dried at 105 °C, and initially weighed before being placed in the climate chambers. The weight of the samples was measured at different time intervals.

The *Ma* value of the samples was calculated according to Equation (5):

(5) $$Ma=\frac{Mt-Mi}{Mi}\times 100,$$

where *Mt*—final weight at different time intervals, *Mi*—initial weight before placing them in climate chambers.

#### 2.4.2. Folding Strength

Fold strength was evaluated using the method described by Sharmilla et al. [41], used to assess the decrease in film elasticity under extreme storage conditions. The number of double folds required for the film to break is defined as the fold strength and evaluated using Equation (6):

(6) F=log 10 D,

where *F*—folding strength, *D*—number of double folding.

The depreciation of CSH film hygroscopicity and elasticity was evaluated by analyzing the results using a stability test based on a regression model and a shelf-life test based on a reliability statistics model (Minitab 20 statistics software. Minitab LLC, State College, PA, USA).

### 2.5. Biodegradability Property: Soil Burial Test

Biodegradability was evaluated by assessing the effects of the natural environment and soil microorganisms on film weight during the soil burial test.

The CSH films obtained were subjected to a 30-day soil burial test using topsoil as a microbial source to simulate the degradation process in the natural environment, using the protocols described by Nissa et al. [1] with minor modifications. The test was conducted at a room temperature of 23 °C ± 2 and under controlled humidity conditions. Three specimens of CSH film samples (3 × 3 cm) were buried at a depth of approximately 2 cm in darkened pots (6.5 × 6.5 × 6.5 cm) filled with topsoil (approximately 1000 g). Polyethylene film samples (3 × 3 cm) were used as positive controls.

The pots were sprayed twice daily to maintain soil moisture and simulate natural conditions. At regular intervals (at six-day intervals), the samples were carefully removed from the pots, gently brushed off, washed three times with distilled water, and dried at room temperature. Biodegradation was evaluated by FTIR analysis and SEM characterization, visual and microscopic observation, and weight loss.

The degree of degradation evaluated by weight loss was calculated using Equation (7):

(7) $$Weight\;loss=\frac{Wi-Wtn}{Wi}\times 100,$$

where *Wi* is the initial weight of the samples and *Wtn* is the weight of the samples at time *tn* where *n* (0…5).

#### 2.5.1. Visual Appearance

The physical changes of the CSH films during the soil burial test were photographed with a camera at least five times during the soil burial test (at six-day intervals). Before photographing, the CSH film samples were removed from the soil, washed, and dried.

#### 2.5.2. Polarized Light Microscopy

The surface of the CSH films was observed with a Nikon Eclipse E100 light microscope (Nikon Corporation, Kanagawa, Japan) and analyzed at least five times (at six-day intervals) during the soil burial test. After washing the samples with distilled water, images of dried CSH film samples (×10 and ×40 magnification) were taken using a Canon PowerShot A640 camera (Melville, NY, USA).

#### 2.5.3. SEM Characterization

Microstructures of the cross-sections and surface morphology were visualized using SEM characterization with a Hitachi TM4000 plus II equipped with a BSE detector and vacuum conductor at an accelerating voltage of 5–15 kV. Digital SEM images of the surface and cross-sections of the CSH films were also analyzed at least five times (at 6-day intervals) during the burial test. The CSH film samples were placed on an SEM tube with carbon tape and the images were recorded (×35, ×500 magnification).

#### 2.5.4. FTIR Analysis

FTIR spectra were acquired using a spectrometer (Bruker, Germany), model Tensor 27 with a ZnSe ATR (Attenuated Total Reflection) accessory The CSH films were tested to investigate the effects of biodegradation on the IR spectrum or changes in molecular structure, analyzed at least five times (at 6-day intervals) during the burial test.

The CSH film samples were washed with distilled water, dried at 30 °C, and crushed for further IR analysis. The analyzed samples were directly spread on the ATR-ZnSe crystal. The ATR crystal was cleaned with ethanol before each measurement to eliminate the presence of residues. FTIR spectra were recorded in the wavelength range of 650–4000 cm^−1^ with 32 scans per sample, at a resolution of 4 cm^−1^ and a speed of 0.32 cm/s.

### 2.6. Statistical Analysis

Data were analyzed with Minitab 20 statistical software using Tukey’s comparison test to detect significant differences (*p* < 0.05). Regression and reliability statistical models were performed to process the data and analyze the influence of different temperatures and RH conditions on the durability and stability of the CSH film. All results were reported as mean ± standard deviation of at least three measurements (*n* = 3).

## 3. Results and Discussion

### 3.1. Physical and Hygroscopic Properties

#### 3.1.1. Film Thickness, *Mc*, *Sd*, and *Tsm*

The hygroscopic properties of the obtained starch-based films are shown in Table 1.

The obtained CSH films showed a lower *Mc* value (19.15 ± 0.020%) and *Tsm* (4.75 ± 0.030%) than the control film, and a higher −*Sd* (60.3 ± 0.025%). For all analyses, there was a statistically significant difference (*p* < 0.05), which was denoted by different lowercase letters in the same column.

This may be due to the incorporation of hydrolyzed starch, which may be more stable and balanced in terms of amylose and amylopectin content following enzymatic hydrolysis, which might have a direct influence on the ability of the film to retain water.

The obtained results are similar to those of the Janik et al. study [21] for the starch film. Rodriguez et al., 2014 [42] observed, for the chitosan films, that the amount of glycerol had a substantial impact on the swelling behavior of the film.

Since the CSH film samples were obtained with the same amount of glycerol as the control sample, but we have a higher *Sd* value for these film samples, lower glycerol ratios can be tested to obtain the film from hydrolyzed starch.

In many applications, such as food packaging, solubility is a desirable property, *Tsm* value being an important factor in selecting films for packaging. However, water resistance and integrity are required for foods with high moisture content, and the high solubility of a film is detrimental.

Low solubility in water is desired in our situation. These results are better than earlier research made by Guo et al. [43], which has shown that pure polysaccharide-based films are almost entirely soluble. The CSH film samples have better qualities than the control sample for applications in food packaging, especially for hygroscopic properties such as *Mc* and *Tsm*, which had lower values.

#### 3.1.2. *WVP*

**Figure 1:**
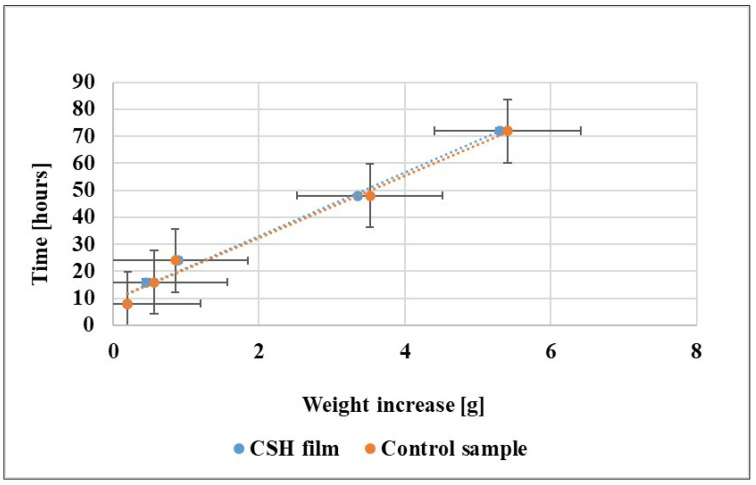
Weight increase plot as a function of time.

Figure 1 and Table 2 present the obtained values of *WVP* for the CSH film sample compared with the control sample.

Compared to the control samples, the values for *WVP* of the CSH film samples are improved but significantly different (using the Tukey comparison test for *p* < 0.05), and similar to the values obtained by Liu Fei et al. [44] for gums films. For certain types of food products, such as processed foods, it is desirable to decrease the WVP of the packaging.

The food packaging material should not facilitate the moisture transfer between the food and the environment for extending the shelf life of the product.

In this context, the addition of oils can improve the *WVP* values of the CSH film samples.

Rodriquez et al. [35] obtained a highly synergistic interaction between glycerol and emulsifiers in potato starch films, achieving the smallest *WVP* results for starch films.

### 3.2. Accelerated Shelf Life and Stability Testing

#### 3.2.1. ASLT

ASLT was used to estimate the time until failure in terms of hygroscopicity and elasticity depreciation of CSH film samples using accelerated temperatures and different RH conditions.

This test was used to estimate the time until 5% of the CSH film samples were expected to fail under normal temperature conditions.

**Figure 2:**
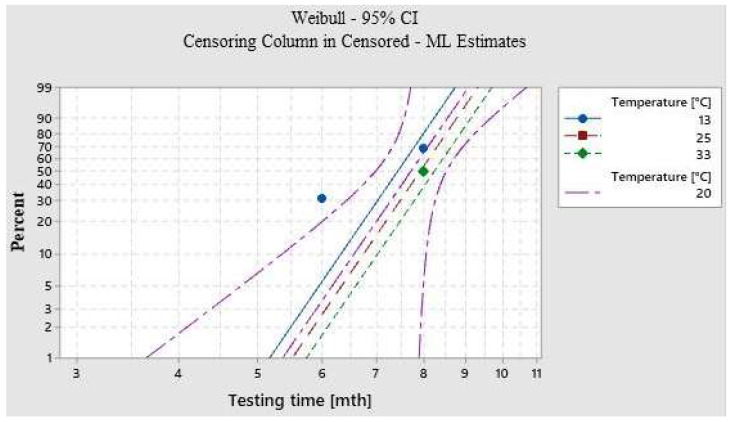
Probability plot (Fitted Arrhenius) for a testing time in months [mth].

Figure 2 and Table 3 show the failure probability plot at three different temperatures and RH conditions during the 60-day test, as well as the predicted failure time under normal temperature and RH conditions.

The “ALT” function (Minitab 20 statistics software) was used to estimate the shelf life at different storage temperatures, taking into account all the parameters analyzed.

If, during the test (T0…T4), any of the parameters are not within the acceptance limit (the sample is severely degraded), this sample is classified as non-compliant and given a score of 0.

In cases where the product has not suffered deterioration of the analyzed parameters, it is given a score of 1, i.e., compliant (data not shown).

The experimental results are processed, specifying the distribution of the data (lognormal, Weibull, etc.) and the mathematical probability model (linear or nonlinear). Since the sample depreciation was affected by temperature and humidity, the linear model is chosen and the Arrhenius equation is used with an error of 5%.

Based on results obtained with Minitab 20 statistical software, 95% of CSH film samples are expected to remain unchanged after 8.89 months when the film is used at normal temperature conditions (20 ± 5 °C and 45 ± 5% RH), with a lower limit of 7.66 months and an upper limit of 10.25 months when temperature and RH change beyond these values.

#### 3.2.2. Stability Test

In order to use the “stability” function in the Minitab 20 statistical software, it is necessary to specify a property that is most important for the stability of the sample under study.

For the CSH film samples, the most important property in terms of hygroscopicity was moisture absorption under various relative humidity conditions (data not shown).

**Figure 3:**
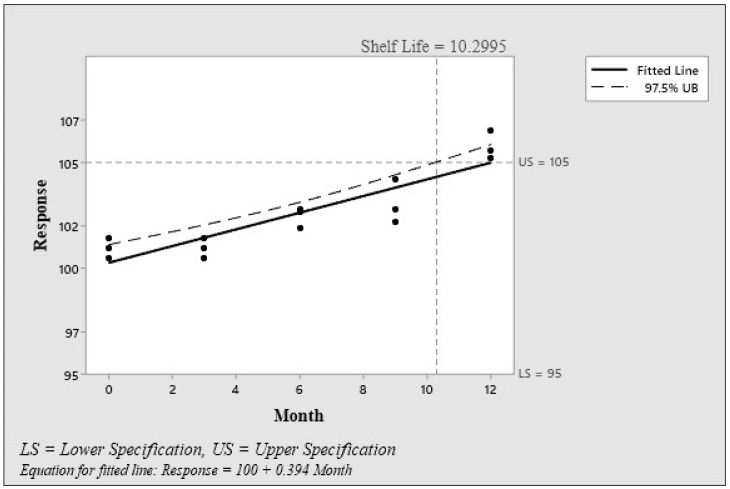
Shelf-life plot.

As part of this function, it is necessary to select the minimum and maximum limits within which the product or film being analyzed must fall to be optimal (Figure 3).

The function checks whether the starting point of the fitted line is between specifications, and then determines the direction of the slope of the fitted lines before deciding from which limit to calculate the shelf life.

If the decrease in average response is statistically significant, the shelf life is determined based on the lower limit of the specification. If the increase in average response over time is statistically significant, the shelf life is determined based on the upper limit of the specification. In terms of design, if the slope of the average line response has a decreasing trend, we should take a look at the upper limit. The general shelf life of CSH film from a hygroscopic point of view was determined to be 10.29 months.

The results of the stability and acceleration tests were in agreement, with the CSH film having an expected shelf life of at least 7.66 months when kept under normal environmental conditions.

### 3.3. Biodegradability Property

**Figure 4:**
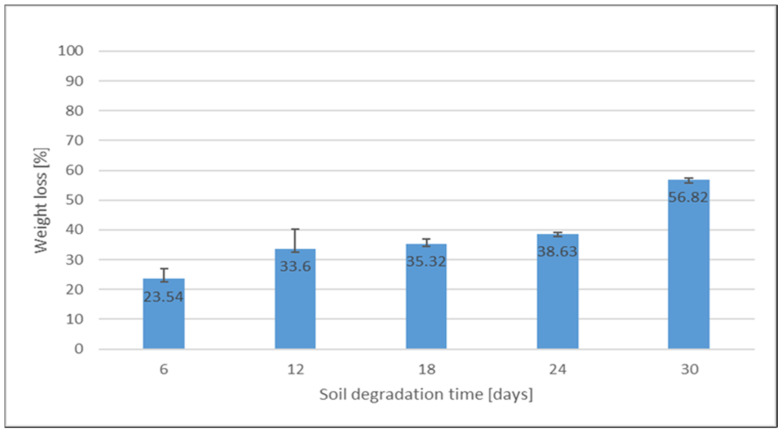
Percentage weight loss of CSH film samples during soil burial test.

The weighted loss plot function of the degradation of the CSH films over time during the burial test is shown in Figure 4.

Biodegradability was evaluated by weight loss during the 30-day soil burial test. The CSH film samples showed a higher percentage of film biodegradability compared to the control samples, which showed little color change but no weight loss.

As expected, significant mass loss was observed, which increased with the duration of burial in the soil. After the 30-day burial test, the CSH films showed a weight loss of 56.82%, which is in agreement with the results of the study by Lucchese et al. [2] and the requirements of the European standard EN13432 [45] (biodegradable plastics must lose 90% of their mass after at least six months and decompose into water, CO_2_ and biomass). As only small CSH film samples were investigated, the obtained results should be considered estimates.

#### 3.3.1. Visual Appearance

**Figure 5:**
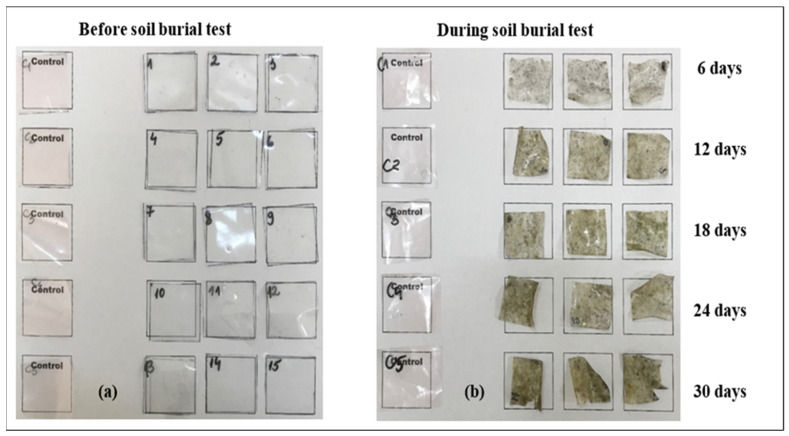
The physical appearance of CSH film samples and control samples before (**a**) and during (**b**) the soil burial test.

Changes in the physical appearance of the CSH film samples due to biodegradation during the soil burial test were monitored and observed by photographing the samples. The photographs of the CSH film samples before and after the biodegradation test are shown in Figure 5.

There was significant degradation by microorganisms in the CSH film samples. The main indicators of biodegradation can be seen in the physical changes of the CSH film samples, which had lost their structural integrity and original appearance and had become glassy and brittle.

#### 3.3.2. Polarized Light Microscopy

Film solubility is an important factor in biodegradability and is directly related to degradation by microorganisms in the soil. At the beginning of the process, changes in film surface and roughness are to be expected due to enzyme attack on the polymer structure. Higher solubility means that the components of the CSH film are more accessible for degradation by the microorganisms present in the soil, such as bacteria, fungi, and protozoa [4].

**Figure 6:**
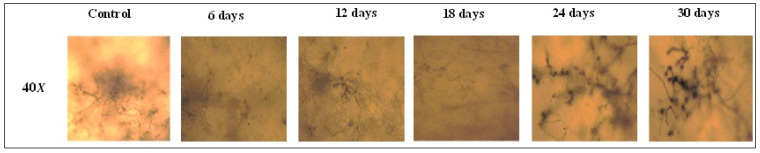
Microscopic images of the CSH of samples during the soil burial test.

The microscopic images of the CSH film samples and control sample (Polyethylene film), recorded during the soil burial test, are presented in Figure 6.

Figure 6 shows an intensification of soil microorganism activity on the surface of the CSH films during the biodegradation test, which is a clear sign of the continuous biodegradation process.

#### 3.3.3. SEM Characterization

**Figure 7:**
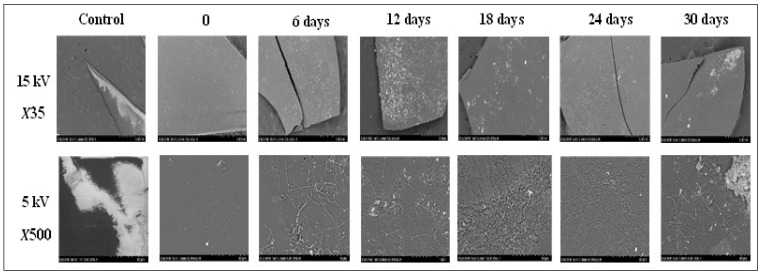
The surface morphology for CSH film samples and control sample (Polyethylene film) before and during the soil burial test.

Bulleted lists look like this: SEM images were also recorded for every test time interval to better understand the biodegradation behavior of the CSH film sample. Figure 7 shows SEM images of the CSH film samples during the soil burial test.

As shown in Figure 7, the surface of the CSH film sample was smooth, compact, and clean before the burial test. During the soil burial test, all CSH film samples lost their original appearance and structural integrity, and the film surface became rough and had small voids. After 30 days of burial test, the surfaces of all CSH film samples exhibited similar microstructure, becoming both dull and porous, with numerous voids and cracks, compared to the control sample, which had no visible surface changes. SEM images confirmed the continuous biodegradation rate, which showed a significant increase in the degradation of CSH films in the soil, mainly by microorganisms.

#### 3.3.4. FTIR Analysis

**Figure 8:**
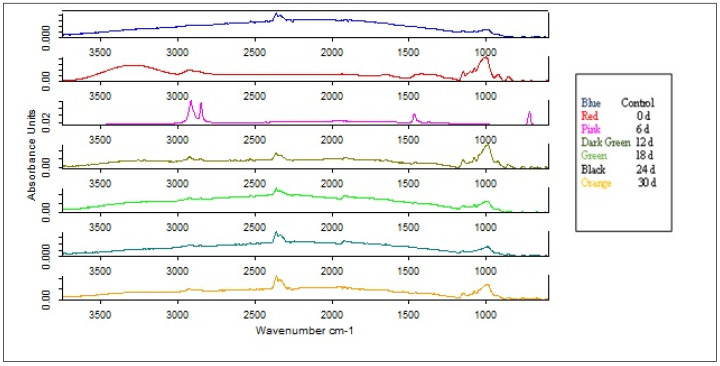
FTIR spectra of CSH film samples and control sample (Polyethylene film) before and during soil burial test.

Using the FTIR spectrum of the CSH film samples during the soil burial test, the changes in molecular structures were investigated. The fingerprint area and characteristic bands of the CSH film and control samples were recorded before and during the soil burial test. Figure 8 shows the FTIR spectra of the CSH film samples during different degradation phases compared to the control sample.

The absorption maximum of the CSH film samples is observed at 3278 cm^−1^ and is attributed to the stretching vibrations of starch due to the presence of hydroxyl groups. The peak attributed to CH groups in the disaccharide bonds is observed in the range 2926–2847 cm^−1^. Most of the absorption peaks are observed in the ranges 1460 cm^−1^, 1150 cm^−1^, 1001 cm^−1^, 924 cm^−1^, and 860 cm^−1^ and are attributed to the CO bonds. After exposing the CSH film samples to the soil, a decrease in the peaks representative of the starch film is observed, indicating a weakening of the bonds. Considering that 56.82% of the CSH film samples were degraded in terms of weight loss, this was also reflected in the FTIR spectrum of the samples as most of the bonds appeared to be cleaved and a decrease and shift in the peaks were observed.

For example, due to the degradation of starch and/or glycerol by soil microorganisms, the OH-stretch band in soil for the degraded CSH film samples not only showed a significant decrease in intensity but also shifted from 3278 cm^−1^ to 2420 cm^−1^ at the end of the biodegradability test. In contrast, the control sample remained undamaged during the soil burial test, and there were no visible changes in the FTIR spectrum.

## 4. Conclusions

The CSH film will remain stable from its hygroscopic qualities for at least 7.66 months if kept in a normal atmosphere, according to the findings of the accelerated shelf life and stability tests.

All analyses conducted during the soil burial test backed up the conclusion that the CSH films underwent a sequentially progressing biodegradation process. This process included weight loss microbial activity and an increase in the film’s porosity, with numerous voids and cracks.

The breakage of the starch component by soil microorganisms, leakage of the plasticizer, and the breakdown of the chemical structure into small molecular units were also observed, as indicated by the SEM micrographs and FTIR analyses.

### Outlook

Biodegradable corn starch hydrolysate films are an excellent choice for dried foods packaging due to their good shelf life at room temperature, which is beneficial when shipping and storing products. They are also environmentally friendly because they are made from renewable plant-based materials and degrade over time, so less waste ends up in landfills. It should be noted, however, that these films are not suitable for foods with high moisture content, as they decompose easily under these conditions. Additionally, when using these films for packaging, care should be taken to ensure that the shelf life of the product is not affected.

## Figures and Tables

**Table 1:** The hygroscopic properties of the CSH film samples compared with the control samples.

Sample	Property			
	Film Thickness [mm]	*Mc* [%]	*Sd* [%]	*Tsm* [%]
Control	0.1756 ± 0.0004 ^a^	23.30 ± 0.025 ^a^	56.2 ± 0.050 ^a^	5.65 ± 0.030 ^a^
CSH film	0.1563 ± 0.0007 ^b^	19.15 ± 0.020 ^b^	60.3 ± 0.025 ^b^	4.75 ± 0.030 ^b^

All results were expressed as mean value ± standard deviation of at least three measurements (*n* = 3). A statistically significant difference (*p* < 0.05) is denoted by different lowercase letters in the same column.

**Table 2:** The results obtained for *WVP*.

Sample	Property		
	R^2^	Y	*WVP*
			[g × mm/m^2^ × h × kPa]
Control	0.9852	11.469x + 9.4239	0.3596 ± 0.0013 ^a^
CSH film	0.9884	11.751x + 9.6306	0.3296 ± 0.0003 ^b^

All results were expressed as mean value ± standard deviation of at least three measurements (*n* = 3). A statistically significant difference (*p* < 0.05) is denoted by different lowercase letters in the same column.

**Table 3:** The results obtained for the estimated time until the failure of the samples.

Unchanged Samples [%]	Time until Degradation [mth]	Expected Durability [mth]	
		Lower Limit	Upper Limit
1	5.36	3.64	7.89
2	5.69	4.09	7.93
3	5.90	4.59	7.95
4	6.05	4.76	7.97
5	6.17	4.91	7.99
6	6.27	5.03	8.00
7	6.35	5.15	8.02
8	6.43	5.25	8.03
9	6.50	5.34	8.04
10	7.00	6.00	8.05
20	7.29	6.42	8.16
30	7.51	6.73	8.39
40	7.77	6.98	8.53
50	7.90	7.17	8.77
60	8.09	7.32	8.94
70	8.29	7.45	9.23
80	8.55	7.57	9.67
90	8.75	7.63	10.03
95	8.89	7.66	10.25
99	9.08	7.71	10.70

## Data Availability

No new data were created or analyzed in this study. Data sharing is not applicable to this article.
